# Relevancia de una guía en quechua sobre uso racional de medicamentos en estudiantes andinos de ciencias de la salud en Perú

**DOI:** 10.15446/rsap.V25n3.99735

**Published:** 2023-05-01

**Authors:** Nancy V. Castilla-Torres, Nicolás Cuya-Arango, Emilio G. Ramírez-Roca

**Affiliations:** 1 NC: Q.F. M. Sc. Atención Farmacéutica y Farmacia Clínica. M. Sc. Gerencia en Servicios de Salud. Ph.D. Salud Pública. Escuela Profesional de Farmacia y Bioquímica, Facultad de Ciencias de la Salud, Universidad Nacional de San Cristóbal de Huamanga. Ayacucho, Perú. nancy.castilla@unsch.edu.pe Universidad Nacional de San Cristóbal de Huamanga Facultad de Ciencias de la Salud Universidad Nacional de San Cristóbal de Huamanga Ayacucho Peru nancy.castilla@unsch.edu.pe; 2 NC: Prof. M. Sc. Docencia en Educación Superior. Ph. D. Ciencias de la Educación. Facultad de Ciencias de la Educación, Universidad Nacional de San Cristóbal de Huamanga. Ayacucho, Perú. nicolas.cuya@unsch.edu.pe Universidad Nacional de San Cristóbal de Huamanga Facultad de Ciencias de la Educación Universidad Nacional de San Cristóbal de Huamanga Ayacucho Peru nicolas.cuya@unsch.edu.pe; 3 ER: Q.F. M. Sc. Bioquímica. M. Sc. Gerencia en Servicios de Salud. Ph.D. Farmacia y Bioquímica. Escuela Profesional de Farmacia y Bioquímica, Facultad de Ciencias de la Salud, Universidad Nacional de San Cristóbal de Huamanga. Ayacucho, Perú. emilio.ramirez@unsch.edu.pe Universidad Nacional de San Cristóbal de Huamanga Facultad de Ciencias de la Salud Universidad Nacional de San Cristóbal de Huamanga Ayacucho Peru emilio.ramirez@unsch.edu.pe

**Keywords:** Utilización de medicamentos, conocimiento, estudiantes, salud *(fuente: DeCS, BIREME)*, Drug utilization, knowledge, students, health *(source: MeSH, NLM)*

## Abstract

**Objetivo:**

Elaborar una guía en quechua sobre uso racional de medicamentos y evaluar su relevancia en el nivel de conocimientos de estudiantes andinos de ciencias de la salud mediante intervención educativa.

**Métodos:**

Estudio cuantitativo, prospectivo, aplicativo y cuasiexperimental con pre-test y post-test de grupo único. La relevancia de la guía en quechua se analizó con las pruebas T-Student, T-Wilcoxon, Kruskall-Wallis y el test de McNemar, mientras que la magnitud del efecto se analizó mediante la correlación biserial (r_b_), d de Cohen y épsilon cuadrado (Ɛ^2^).

**Resultados:**

Participaron en el estudio 115 estudiantes, 96 (83,5%) de ellos mujeres. Luego de la intervención educativa, el nivel de conocimiento se elevó de nivel bajo a alto; la frecuencia de alumnos aprobados aumentó; el análisis intragrupal demostró incremento en el promedio de calificación de los estudiantes de las tres escuelas, de 10,8±2,3 pre-test a 14,5±3,2 post-test (p-valor=0,001), con magnitud de efecto alto (r_b_ >0,5), principalmente en las escuelas de Farmacia y Obstetricia. A nivel intergrupal también se halló incremento entre las calificaciones de las tres escuelas con tamaño de efecto grande (χ^2^=11,9; gl=2; p-valor=0,003, Ɛ^2^=0,11), especialmente entre los estudiantes de las escuelas de Farmacia y Enfermería (p-valor=0,009), así como entre Obstetricia y Enfermería (p-valor=0,002).

**Conclusión:**

La guía en quechua, compuesta por tres módulos: automedicación, uso racional de medicamentos, antibióticos y resistencia bacteriana, fue relevante porque incrementó el nivel de conocimientos de los estudiantes de las tres escuelas.

## Abstract (en)

**Objective:**

To elaborate a guide in Quechua on rational use of medicines and evaluate its relevance in the level of knowledge of Andean health science students. To develop a guide in Quechua on the rational use of medicines and evaluate its relevance in the level of knowledge of Andean health science students through educational intervention.

**Methods:**

A quantitative, longitudinal, prospective, applicative, quasi-experimental study with single-group pretest and posttest. The relevance of the guide in Quechua was analyzed with Student's t-test, Wilcoxon's t-test, Kruskall-Wallis and McNemar's test, while the magnitude of the effect by means of biserial correlation (r_b_), Cohen's d and epsilon squared (Ɛ^2^).

**Results:**

A total of 115 students participated in the study, mostly 96 (83.5%) females; after the educational intervention the level of knowledge rose from low to high level, the frequency of passing students increased, the intra-group analysis showed increase in the average grade of the students of the 03 schools, from 10.8±2.3 pre-test to 14.5±3.2 post-test, (p-value =0.001), with high effect magnitude (r_b_>0.5), mainly in the schools of Pharmacy and Obstetrics. Mainly in the schools of Pharmacy and Obstetrics. At the inter-group level, increase was also found among the grades of the 03 schools with large effect size (χ^2^=11.9; gl=2; p-value = 0.003, Ɛ^2^= 0.11), especially among the students of the schools of Pharmacy and Nursing (p-value =0.009), as well as between Obstetrics and Nursing (p-value =0.002).

**Conclusion:**

The guide in Quechua composed of 03 modules: self-medication, rational use of medicines, antibiotics and bacterial resistance was relevant because it increased the level of knowledge of the students of the three schools.

El quechua y sus variantes se distribuyen en siete regiones de América del Sur, entre ellas Perú, Ecuador, Colombia, Argentina, Chile y Brasil 1. Perú es un país pluricultural y multilingüe, con más de 3 799 780 usuarios 2, de los cuales un número importante son estudiantes universitarios de las carreras de ciencias de la salud que requieren estrategias de aprendizaje intercultural para poder comunicar e informar a pacientes quechua-hablantes con respecto a sus enfermedades, su farmacoterapia y el uso racional de los medicamentos.

La promoción del uso racional de medicamentos (URM), desde una óptica integral, es responsabilidad de todos; es decir, del prescriptor, del dispensador, del fabricante, del usuario final, de la comunidad científica y de la autoridad sanitaria 3. Para alcanzar este objetivo, es imprescindible contar con mejores estrategias de comunicación en los servicios de atención de salud, además de un personal de salud bien capacitado, con la mejor disposición para brindar atención e información en su respectiva lengua indígena u originaria, considerando la inclusión de la interculturalidad, sobre todo en Perú, país con una gran cantidad de población quechua que es la menos atendida 4-6.

En el Perú se han dado casos de discriminación, intolerancia y desatención de los usuarios de lenguas originarias, como el quechua y el aimara, en sectores como el judicial y de salud 4,6. Según reportes de la I Encuesta Nacional sobre Percepciones y Actitudes sobre Diversidad Cultural y Discriminación Étnico-Racial, la encuesta reportó que el 22% de los encuestados se sintieron discriminados en un establecimiento de salud, el 16% por su forma de hablar y el 6% por el idioma o la lengua que habla, mientras que un 59% percibió discriminación a la población quechua y aimara por los factores mencionados anteriormente, sumándole a ello el factor de la indumentaria 7.

Los tipos frecuentes de uso irracional de medicamentos son la polifarmacia, el uso inadecuado de antibióticos y la automedicación 8.

La morbimortalidad relacionada con el uso irracional de los medicamentos es un problema de salud pública 7 con importantes consecuencias, como resistencia bacteriana, reacciones adversas, errores de medicación, desperdicio de recursos, pérdida de confianza del paciente, entre otras 9, y evitarla depende en gran parte del personal de salud 10,11.

Para poder optimizar el uso racional de los medicamentos se requiere un conjunto de acciones de comunicación, educación e información, con el objetivo de alcanzar actitudes y conductas acordes con la problemática, labor que se debe iniciar en las aulas universitarias 12,13.

En Ayacucho, un gran porcentaje de los estudiantes universitarios de las carreras de ciencias de la salud son quechua-hablantes. Por ello, es necesario complementar su educación en su propio idioma, especialmente con respecto a los medicamentos, que requieren indicaciones y palabras específicas no muy comúnmente utilizadas en su comunicación diaria, pero que son necesarios para una mejor información.

Se requieren materiales de educación sobre medicamentos con información necesaria y con términos definidos en quechua para mejorar su comunicación con los pacientes quechua-hablantes. Esta es una labor que se inicia desde el pregrado en los diversos establecimientos de salud y posteriormente como profesionales de salud que se inicia en el Servicio Rural y Urbano Marginal de Salud (<<).

Por todo lo antes expuesto, se planteó el objetivo de elaborar una guía en quechua sobre uso racional de medicamentos y evaluar su relevancia en el nivel de conocimiento de estudiantes andinos de las carreras de ciencias de la salud de la Universidad Nacional de San Cristóbal de Huamanga (UNSCH), en Ayacucho, Perú.

## MÉTODOS

Diseño cuantitativo, longitudinal, prospectivo, aplicativo y cuasiexperimental con pre-test y post-test de grupo único. Se reclutó a un total de 115 estudiantes, integrado por 35 estudiantes de enfermería, 42 de obstetricia y 65 de farmacia y bioquímica de la Facultad de Ciencias de la Salud de la UNSCH, obtenidos mediante muestreo no probabilístico intencional. Se incluyó solo a estudiantes de los dos últimos años, que entienden y leen el idioma quechua, que hubieran cursado la asignatura de farmacología y estuvieran matriculados en los semestres 2020 y 2021, presentes en el horario de la intervención. Se excluyó a estudiantes de los primeros ciclos, debido a que aún no tienen formación con respecto a medicamentos. El marco muestral de lista estuvo constituido por los alumnos matriculados en la Facultad de Ciencias de la Salud por cada escuela mencionada. Así mismo, se seleccionó el horario de la asignatura al que más alumnos convergieran, el cual previamente estuvo programado por la unidad de estadística de la UNSCH para cada docente, por lo que se solicitó a los docentes que nos cedieran dos horas de su clase para la investigación.

Se realizó un estudio piloto para determinar la confiabilidad del instrumento mediante la fórmula de Kuder Richardson, con el que se obtuvo un valor de 0,8. Se validó el instrumento por juicio de expertos y se obtuvo un coeficiente de V de Aiken de 0,9.

**Figura 1:**
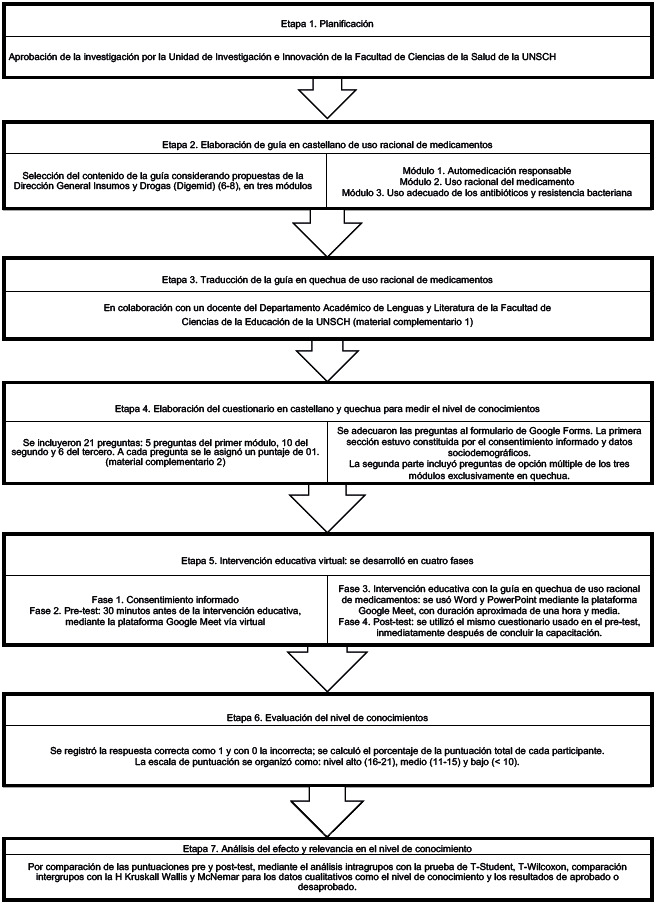
Secuencia de la investigación

La investigación se llevó a cabo en siete etapas (Figura 1). La intervención educativa y la evaluación del nivel de conocimientos a través del pre y el post-test se realizó vía virtual, debido a la imposibilidad de hacer clases presenciales por la pandemia causada por la COVID-19.

### Aspectos éticos

La investigación fue aprobada por el comité de ética de la UNSCH (certificado N°3-VRI-UNSCH) y por el Instituto de Investigación e Innovación de la Facultad de Ciencias de la Salud (RD-N°051-2020-FCSA/D) y todos los participantes dieron su consentimiento informado. Los datos procesados se conservaron en forma anónima, considerando los principios de la Declaración de Helsinski.

### Análisis estadístico

La base de datos se procesó, ordenó y codificó utilizando la hoja de cálculo Microsoft Office Excel. Para el análisis estadístico se utilizó el programa Jamovi 2020 y el paquete estadístico IBM SPSS (Statistical Package for Social Sciences) versión 26 para Windows.

Se verificó la distribución normal con la prueba de Kolmogorov-Smirnov y a través de los índices de asimetría (g1) y curtosis (g2). Los resultados descriptivos de las variables se tabularon en tablas de doble entrada, considerando sus dimensiones e indicadores en escala numérica (de razón o intervalo) y en escala categórica (nominal y ordinal) con la prueba de Chi cuadrado (x^2^), la prueba de Pearson y asociación lineal por lineal para p-valor ≥0,05.

El análisis inferencial, para determinar la relevancia de la guía en quechua mediante la intervención educativa, se midió evaluando el nivel del conocimiento, el número de aprobados y la diferencia de los promedios de las calificaciones pre-test y post-test, intergrupos e intragrupos de los estudiantes de las tres escuelas en las tres áreas del uso racional de medicamentos.

En el análisis intragrupal se empleó la prueba T-Wilcoxon para muestras relacionadas con distribución no normal. Para la magnitud del efecto en la forma de correlación biserial (r_b_) 14 se tuvieron en cuenta los valores de rg <0,1 (insignificante), entre 0,1 y 0,3 (bajo), entre 0,3 y 0,5 (moderado) y <0,5, (alto). Asimismo, se utilizó la prueba de T-Student para muestras relacionadas con distribución normal y la prueba de Cohen 15 para medir la magnitud del efecto en función de su valor <0,2 (insignificante), entre 0,2 y 0,5 (pequeño), entre 0,5 y 0,8 (moderado) y >0,8 (grande).

La comparación de las puntuaciones intergrupales de las tres escuelas se llevó a cabo mediante el H Kruskall Wallis para más de dos muestras independientes, en tanto que para la significancia práctica de los resultados se empleó el estadístico de tamaño del efecto épsilon cuadrado (Ɛ^2^)_:_ 0,01, pequeña; 0,06, mediana; y 0,14, grande 16.

Para las variables categóricas, como el nivel de conocimiento y los resultados del número de aprobados o desaprobados, se empleó la prueba de McNemar, en todos los casos con un nivel de confianza del 95%.

## RESULTADOS

Del estudio, de 115 estudiantes se destacaron 96 (83,5%) mujeres, en su mayoría de 20 a 24 años; 52 (45,2%) pertenecían a la Escuela Profesional de Farmacia y Bioquímica, 40 (34,8%) a Enfermería y 23 (20,0%) a Obstetricia. La mayoría procedía del área rural y dependía de sus padres (Tabla 1).

**Tabla 1:** Perfil socio-demográfico de estudiantes andinos de ciencias de la Salud

Perfil sociodemográfico	Escuelas				
	Farmacia y Bioquímica 52(45,2)	Obstetricia 40(34,8)	Enfermería 23(20,0)	Total (n=115)	*P*
	n(%)	n(%)	n(%)		
Edad (años)					0,221^*^
19 - 21	3(2,6)	14(12,2)	2(1,7)	19(16,5)	
22 - 24	26(22,6)	20(17,4)	11(9,6)	57(49,6)	
25 - 28	23(20,0)	6(5,2)	10(8,7)	39(33,9)	
Género					0,000^**^
Femenino	34(29,6)	40(34,8)	22(19,1)	96(83,5)	
Masculino	18(15,7)	0(0,0)	1(0,9)	19(16,5)	
Dependencia económica					0,417^*^
Padre	7(6,1)	2(1,7)	3(2,6)	12(10,4)	
Madre	5(4,3)	4(3,5)	2(1,7)	11(9,6)	
De ambos padres	28(24,3)	26(22,6)	15(13,0)	69(60,0)	
Otros	12(10,4)	8(7,0)	3(2,6)	23(20,0)	
Procedencia					0,833^**^
Urbano	26(22,6)	18(15,7)	10(8,7)	54(47,0)	
Rural	26(22,6)	22(19,1)	13(11,3)	61(53,0)	

^*^ x^2^: Chi cuadrado asociación lineal por lineal, p-valor ≥ 0,05; ^**^ x^2^: Chi cuadrado de Pearson, p-valor ≥ 0,05

**Figura 2:**
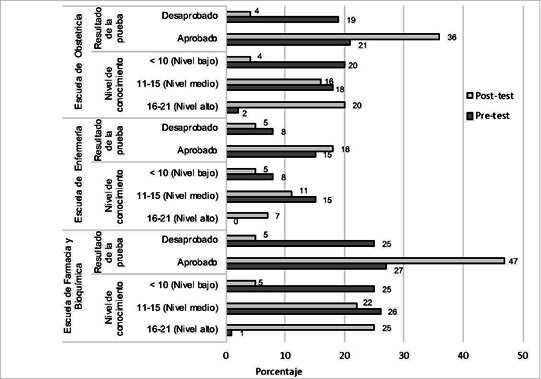
Nivel de conocimientos y frecuencia de aprobados pre y post-test sobre uso racional de medicamentos en quechua en estudiantes andinos de ciencias de la salud

Luego de la intervención educativa con la guía en quechua sobre uso racional de medicamentos, la frecuencia de alumnos aprobados de las tres escuelas se incrementó, en tanto que el nivel de conocimientos mejoró de nivel bajo (<10 puntos) a alto (16 a 21 puntos), principalmente en los estudiantes de las escuelas de Farmacia y Obstetricia (Figura 2).

En el análisis intragrupal se hallaron diferencias significativas entre las medias de las calificaciones de las tres escuelas, que aumentaron de 10,8±2,3 puntos en el pre-test a 14,5±3,2 puntos en el post-test (p-valor = 0,001), con magnitud de efecto alto (rb > 0,5), especialmente en las escuelas de Farmacia y Bioquímica y Obstetricia (Tabla 2).

A nivel intergrupal post-test también se halló un incremento de las calificaciones de las tres escuelas, sobre todo entre los estudiantes de las escuelas de Farmacia y Enfermería (p-valor=0,009), así como entre Obstetricia y Enfermería (p-valor =0,002), cuya magnitud del efecto fue grande (x^2^=11,9; gl=2; p-valor = 0,003, Ɛ^2^= 0,11) (Tabla 3).

**Tabla 2:** Análisis intragrupal pre-test y post-test de estudiantes andinos de ciencias de la salud

Escuelas	Media de la calificación ($\overset{-}{x}$ ± DE)		Diferencia de media	g1	g2	Valor de la prueba	Significancia práctica	p-valor^*^
	Pre-test	Post-test						
Farmacia y Bioquímica	10,7±2,3	14,9±3,3	-4,5±0,6	0,6	2,4	55.0a	-0,9 c	0,001
Enfermería	11,0±2,2	12,4±2,9	-1,4±0,7	-0,7	-0,7	-2,0b	-0,4 d	0,054
Obstetricia	10,7±2,3	15,2±2,7	-5,5±0,5	0,2	0,2	3,0a	-0,9 c	0,001
Total de las 3 escuelas	10,8±2,3	14,5±3,2	-4,0±0,4			334a	-0,9 c	0,001

$\overset{-}{x}$ : Media; DE: Desviación estándar; g_1_ = Asimetría; g_2_ = Curtosis; ^*^p<0,005; ^a^ Wilcoxon; ^b^ T student; ^c^ Rango de correlación biserial (r_b_); ^d^ d de Cohen.

**Tabla 3:** Análisis intergrupos pre-test y post-test de estudiantes de ciencias de la salud

Comparación de pares de escuelas		Pre test						Post test					
		H	p-valor	χ²	gl	p-valor*	ε²	H	p-valor	χ²	gl	p-valor*	ε²
Farmacia	Obstetricia	-0,1	0,999					0,5	0,938				
Farmacia	Enfermería	1,6	0,490	1,3	2	0,523	0,011	-4,2	0,009	11,9	2	0,003	0,11
Obstetricia	Enfermería	1,3	0,636					-4,7	0,002				

H: Prueba de Kruskal-Wallis; χ² Chi cuadrado; ^*^p<0,005; ε² épsilon-cuadrado; gl: grados de libertad.

En la Tabla 4 se evidencia que el nivel de conocimientos mejoró en las tres escuelas, con incremento de la frecuencia de respuestas correctas, sobre todo en la dimensión de automedicación, especialmente en las escuelas de Farmacia y Enfermería. En el módulo de uso racional de medicamentos se observa una mejor respuesta en las escuelas de Farmacia y Obstetricia, mientras que el área de uso adecuado de antibióticos y resistencia bacteriana mejoró solo en el grupo de la escuela de Farmacia y Bioquímica.

**Tabla 4:** Frecuencia de respuestas correctas según tipo de módulo de uso racional de medicamentos de estudiantes andinos de ciencias de la salud

Numero de ítem*	Prueba	Respuestas correctas por escuelas				Porcentaje de cambio de respuestas correctas					
		Farmacia y bioquímica (n/%)	Obstetricia (n/%)	Enfermería (n/%)	p-valor	Farmacia y Bioquímica (n/%)		Obstetricia (n/%)		Enfermería (n/%)	
Automedicación. kikillanmanta hampikuq											
1	Pre-test	5(4,3)	30(26,1)	17(14,8)	0,000	11(9,6)	Mejora	-8(-7)	Empeora	2(1,7)	Mejora
	Post-test	16(13,9)	22(19,1)	19(16,5)	0,000						
2	Pre-test	50(43,5)	32(27,8)	20(17,4)	0,051	2(1,7)	Mejora	6(5,2)	Mejora	-1(-0,9)	Empeora
	Post-test	52(45,2)	38(33,0)	19(16,5)	0,008						
3	Pre-test	13(11,3)	12(10,4)	1(0,9)	0,055	26(22,6)	Mejora	8(7)	Mejora	10(8,7)	Mejora
	Post-test	39(33,9)	20(17,4)	11(9,6)	0,018						
4	Pre-test	31(27,0)	32(27,8)	16(13,9)	0,112	7(6)	Mejora	2(1,8)	Mejora	2(1,8)	Mejora
	Post-test	38(33,0)	34(29,6)	18(15,7)	0,389						
5	Pre-test	40(34,8)	34(29,6)	10(8,7)	0,001	-13(-11,3)	Empeora	-14(-12,2)	Empeora	7(6,1)	Mejora
	Post-test	27(23,5)	20(17,4)	17(14,8)	0,141						
Uso racional de medicamentos. Hampikunanata allinchallata hampina											
6	Pre-test	20(17,4)	18(15,7)	12(10,4)	0,528	-6(-5,2)	Empeora	-4(-3,5)	Empeora	-4(-3,4)	Empeora
	Post-test	14(12,2)	14(12,2)	8(7,0)	0,655						
7	Pre-test	44(38,3)	30(26,1)	21(18,3)	0,227	1(0,8)	Mejora	6(5,2)	Mejora	-5(-4,4)	Empeora
	Post-test	45(39,1)	36(31,3)	16(13,9)	0,084						
8	Pre-test	46(40,0)	30(26,1)	20(17,4)	0,200	0(0)	Sin cambios	4(3,5)	Mejora	0(0)	Sin cambios
	Post-test	46(40,0)	34(29,6)	20(17,4)	0,887						
9	Pre-test	34(29,6)	14(12,2)	5(4,3)	0,000	-1(-0,9)	Empeora	-6(-5,2)	Empeora	1(0,9)	Sin cambios
	Post-test	33(28,7)	8(7,0)	6(5,2)	0,000						
10	Pre-test	24(20,9)	30(26,1)	9(7,8)	0,005	-7(-6,1)	Empeora	-18(-15,7)	Empeora	1(0,9)	Sin cambios
	Post-test	17(14,8)	12(10,4)	10(8,7)	0,536						
11	Pre-test	14(12,2)	18(15,7)	2(1,7)	0,008	18(15,6)	Mejora	6(5,2)	Mejora	5(4,4)	Mejora
	Post-test	32(27,8)	24(20,9)	7(6,1)	0,032						
12	Pre-test	39(33,9)	26(22,6)	21(18,3)	0,069	-9(-7,8)	Empeora	0(0)	Sin cambios	-5(4,4)	Empeora
	Post-test	30(26,1)	26(22,6)	16(13,9)	0,574						
13	Pre-test	16(13,9)	10(8,7)	7(6,1)	0,815	16(13,9)	Mejora	18(15,6)	Mejora	2(1,7)	Mejora
	Post-test	32(27,8)	28(24,3)	9(7,8)	0,005						
14	Pre-test	11(9,6)	18(15,7)	7(6,1)	0,050	18(15,6)	Mejora	8(6,9)	Mejora	-2(-1,8)	Empeora
	Post-test	29(25,2)	26(22,6)	5(4,3)	0,003						
15	Pre-test	17(14,8)	12(10,4)	9(7,8)	0,757	-4(-3,5)	Empeora	-6(-5,2)	Empeora	-4(-3,5)	Empeora
	Post-test	13(11,3)	6(5,2)	5(4,3)	0,501						
Uso adecuado de antibióticos y resistencia bacteriana. Allin antibióticos hampikunapaq chaymanta bacteriana mana wañuq											
16	Pre-test	41(35,7)	34(29,6)	19(16,5)	0,745	4(3,4)	Mejora	-4(-3,5)	Empeora	1(0,9)	Mejora
	Post-test	45(39,1)	30(26,1)	20(17,4)	0,290						
17	Pre-test	39(33,9)	34(29,6)	19(16,5)	0,464	-17(-14,8)	Empeora	-14(-12,2)	Empeora	-1(-0,8)	Empeora
	Post-test	22(19,1)	20(17,4)	18(15,7)	0,015						
18	Pre-test	27(23,5)	22(19,1)	20(17,4)	0,012	14(12,2)	Mejora	12(10,5)	Mejora	-11(-9,6)	Empeora
	Post-test	41(35,7)	34(29,6)	9(7,8)	0,000						
19	Pre-test	40(34,8)	36(31,3)	18(15,7)	0,244	-20(-17,4)	Empeora	-16(-13,9)	Empeora	-2(-1,8)	Empeora
	Post-test	20(17,4)	20(17,4)	16(13,9)	0,045						
20	Pre-test	44(38,3)	28(24,3)	16(13,9)	0,177	3(2,6)	Mejora	8(7)	Mejora	7(6,1)	Mejora
	Post-test	47(40,9)	36(31,3)	23(20,0)	0,294						
21	Pre-test	46(40,0)	38(33,0)	23(20,0)	0,162	5(4,3)	Mejora	0(0)	Sin cambios	0(0)	Sin cambios
	Post-test	51(44,3)	38(33,0)	23(20,0)	0,447						

^*^ ltem: Módulo 1. Automedicación. kikillanmanta hampikuq.

1 y 2: Sobre los riesgos de la automedicación, marque el incorrecto. Kikillanmanta hampikuq wañurunmanmi, mana allinta chakachaykuy.

3: De los siguientes profesionales de la salud, ¿quiénes deben recetar medicamentos?, marque las alternativas correctas. Kay hampiq llamkaqkunapaq ¿Pikunataq recetata qunmanku? Chakataykuy allin nisqanta.

4 y 5: Sobre la automedicación responsable, marque los incorrectos. Kikillanmanta hampikuq, mana allinkunata chakachaykuy. Módulo 2. Uso racional de medicamentos. Hampikunawan allinchallata hampina.

6: Sobre los medicamentos de venta libre, marque los incorrectos. Hampikuna mana rantiy qatipasqa, mana allinkunata chakachaykuy. 7: Sobre el uso racional de medicamentos, marque los incorrectos. Allin hampikunata hampinapaq, mana allinkunata chakachaykuy.

8: ¿Qué debes verificar antes de adquirir un medicamento?, marque los correctos. ¿Imatataq qawanayki manaraq hampita rantispayki? Allinllanta chakataykuy.

9: ¿Qué debes verificar antes de adquirir un medicamento?, marque los incorrectos. ¿Imatataq qawanayki manaraq hampita rantispayki? Mana allinllanta chakataykuy.

10: Información que el paciente debe tener antes de tomar medicamentos, marque lo incorrecto. Manaraq hampikunata upyaspa unquqqa kaykunata yachanman. Chakataykuy mana allinninta (X).

11: Respecto al uso de la receta médica, marque las correctas. Recetamanta allin kasunapaq, Chakataykuy allin nisqanta.

12: Respecto al almacenamiento de los medicamentos, marque lo incorrecto. Hampikunamanta waqaychasqa, chakataykuy allinllanta.

13: Respecto a los medicamentos en el embarazo, marque lo correcto. Hampikunamanta warmi wiksayuqpa, chakataykuy allinllanta.

14: Respecto a los medicamentos para niños y adultos, marque lo correcto de las afirmaciones. Hampikunamanta wawakunapaq chaymanta machukunapaq, Chakataykuy arí, nisqanta, chakataykuy.

15: Respecto al uso de los medicamentos en el anciano, marca las respuestas incorrectas. Machukunapa hampikunamanta, chakataykuy mana allin nisqanta. Módulo 3. Uso adecuado de antibióticos y resistencia bacteriana. Allin antibióticos hampikunapaq chaymanta bacteriana mana wañuq.

16 y 17: Respecto al uso de los antibióticos marque los correctos. Antibioticokuna hapinapaq, chakataykuy.

18: Respecto al uso de los antibióticos, marque la opción correcta. Antibioticoskunamanta imayna hapina, chakataykuy mana allinnin kaqta.

19: Respecto al almacenamiento de los antibióticos, marque lo falso. Antibiótico waqaychanamanta, chakataykuy mana allinninta.

20: ¿Cómo evitar resistencia bacteriana?, marque los incorrectos. ¿Imaynatataq bacteria wañunanpaq ruwachwan?, chakataykuy mana allinninta.

21: Los antibióticos deben comprarse en; marque lo correcto. Antibioticokunata rantina kanman. chakataykuy allinninta.

## DISCUSIÓN

El presente estudio, cuasiexperimental, es pionero en elaborar una guía sobre uso racional de medicamentos en el idioma quechua, así como en medir su relevancia en el nivel de conocimientos de los estudiantes de ciencias de la salud. Son nulos los trabajos con estos objetivos que sirvan de contraste.

En este estudio se halló que todos los participantes hablan, leen, o comprenden el idioma quechua. En contraste, en otra investigación en una universidad nacional del Perú, en la cual sólo se evaluó el nivel de conocimiento en estudiantes de ciencias de la salud, se halló que menos de la mitad manifestó hablar, leer o comprender quechua, pero la mayoría procedía del área urbana 5

El quechua es un lengua indígena con más de tres millones de hablantes en Perú 2, siendo Apurímac, Huancavelica, Ayacucho y Cuzco los departamentos altoandinos con más densidad de usuarios 6,17. A pesar de ello, son escasas las universidades del Perú que consideran a este idioma dentro de su plan de estudios 18,19, y la carencia de instrumentos de enseñanza sobre medicamentos en este idioma incrementa la brecha entre la enseñanza de técnicas de comunicación e información 5.

En Perú, además, se reporta una inadecuada atención a los pacientes que forman parte de la población quechua y aimara 2,5, pese a la existencia de la Ley N.° 29735 que, en su artículo 6.1, enfatiza la obligación de que todo servidor de cualquier entidad pública o privada debe hacer uso de las lenguas indígenas u originarias de manera oral y escrita al atender a los usuarios; asimismo, el artículo 6.2 enfatiza que toda persona tiene derecho a recibir atención e información en su respectiva lengua indígena u originaria en cualquier tipo de entidad que preste servicios públicos 19.

Ante la carencia de recursos de enseñanza en quechua, el médico Lopera publicó el texto titulado *El Manual de semiología en quechua,* una guía para conseguir la historia clínica de pacientes; grandioso aporte para hacer una adecuada anamnesis de los pacientes quechua-hablantes, así como para aprender a convivir y comprenderlos 20, mas no incluye ningún capítulo sobre medicamentos.

La inclusión del idioma quechua en las mallas curriculares de todas las carreras del Perú es de alta prioridad, en especial en ciencias de la salud, debido a las evidentes limitaciones en la comunicación con los pacientes, cuya fisura se hace más plausible en su primer contacto con pacientes quechua-hablantes durante sus prácticas preprofesionales y en su ejercicio profesional 6,20, considerando que los medicamentos son una herramienta fundamental para la prevención, la curación, la atenuación y el tratamiento de las enfermedades y sus síntomas. Sin embargo, cuando estos se utilizan de manera inapropiada o irracional, se convierten en una amenaza para la salud individual y colectiva 3,9. No importa cuán efectivo o seguro sea un sea un fármaco, este solo puede cumplir su función si es utilizado correctamente y con una adecuada información 7.

La intervención educativa mediante la guía en quechua mejoró el nivel de conocimientos de los estudiantes de las tres escuelas en los tres rubros: automedicación, uso racional de medicamentos, de antibióticos y resistencia bacteriana, además de incrementar el promedio de puntuación, con alto impacto práctico, sobre todo en las escuelas de Farmacia y Bioquímica y Obstetricia en el análisis intragrupal. Por su parte, el análisis intergrupal post-test entre las escuelas de Farmacia y Enfermería, así como entre Obstetricia y Enfermería, mostró que la magnitud del efecto post-test fue grande.

Estos resultados son alentadores debido a que según varios estudios la intervención educativa no solo incrementa el nivel de conocimiento, sino que mejora las habilidades de comunicación en diversos aspectos 10,19,20, por lo que a pesar de que los estudiantes entienden el idioma quechua, la guía incrementa el uso de palabras técnicas específicas sobre medicamentos en las tres áreas antes mencionadas que otorgan habilidades para informar a los pacientes que acuden a establecimientos farmacéuticos.

En diversas investigaciones se registran elevados porcentajes de uso irracional de medicamentos; los más reportados son uso inadecuado de antibióticos, resistencia bacteriana, automedicación inadecuada y polifarmacia, problema que se reduciría si la población entendiera sobre el tema y sus implicancias en su lengua materna 8,12.

En este estudio se evidencian progresos en el nivel de conocimientos al mostrar mejoras en las respuestas correctas, en la dimensión de automedicación, especialmente en los estudiantes de las escuelas de Farmacia y Bioquímica y Enfermería. Es imprescindible que los estudiantes de Ciencias de la Salud dominen estos conceptos para que en el futuro lo reflejen en su labor profesional.

La automedicación puede ocasionar riesgos en su salud, como enmascaramiento de la enfermedad, reacciones adversas, interacciones medicamentosas, incremento de la resistencia bacteriana y drogodependencia 11-13,21, que podría evitarse con la adecuada información en su lengua originaria 4-6,8.

Del mismo modo, la investigación evidencia mejoras en el área del uso racional de medicamentos, con incremento en el número de respuestas correctas de los estudiantes de las tres escuelas, destacando las escuelas de Farmacia y Obstetricia, mientras que en la dimensión de uso adecuado de antibióticos y resistencia bacteriana, se mostraron mejores respuestas en la escuela de Farmacia y Bioquímica.

La resistencia bacteriana es consecuencia del uso irracional del antibiótico, que es la capacidad natural o adquirida que tienen las bacterias para resistir a los efectos bactericidas o bacteriostáticos de los antibióticos 22, lo que pone en peligro la eficacia en la prevención y en el tratamiento de una serie de infecciones 23,24, problema es frecuente en países pobres y que empeora cuando los profesionales de salud desconocen el tema 25,26.

El artículo 48 de la Constitución Política del Perú señala que "son idiomas oficiales el castellano y, en zonas donde predomine, también lo son el quechua, el aimara y las demás lenguas aborígenes, según ley".

Por consiguiente, la formación universitaria del profesional de ciencias de la salud, además de las asignaturas de formación específica, debería complementarse con asignaturas en lenguas nativas, especialmente el quechua, utilizando diversos materiales y estrategias educativas para mejorar la relación profesional-paciente, a fin de que la comunicación sea más empática y eficaz 4,6.

Los pacientes atendidos e informados en su lengua originaria tienen una mejor comprensión de sus enfermedades y por lo tanto una actitud más responsable con respecto a sus elecciones de tratamiento farmacológico y no farmacológico 20. Del mismo modo, los pacientes no suelen regresar a un establecimiento de salud si perciben ser mal atendidos o no haber entendido la información brindada por el profesional de la salud 5,18.

En Perú existe un gran porcentaje de estudiantes universitarios de ciencias de la salud quechua-hablantes, por lo que necesitan complementar su educación en su idioma materno con materiales complementarios sobre medicamentos, con términos precisos traducidos al idioma quechua, para mejorar su comunicación con los pacientes quechua-hablantes.

Como se puede evidenciar en los resultados, la relevancia de la guía en quechua se reveló en la mejora del nivel de conocimientos sobre uso racional de medicamentos en las tres áreas, lo que se demuestra con el incremento de promedio de las calificaciones obtenidas y el número de aprobados de los estudiantes de ciencias de la salud de la UNSCH.

Es importante destacar las fortalezas de la investigación, que son el dominio del idioma quechua de los autores y del tema de uso racional de medicamentos en el ámbito hospitalario y la docencia universitaria, pero también se debe informar algunas limitaciones del estudio dado que la intervención educativa se realizó de modo virtual, utilizando la plataforma Google Forms y Google Meet, ya que inicialmente se concibió para su aplicación en las aulas universitarias de modo presencial, lo cual no se concretó debido a la pandemia del COVID-19. Pese a ello, se logró obtener satisfactoriamente los objetivos planteados.

Por todo lo anteriormente descrito, la aplicabilidad de la guía en quechua en primera instancia será durante las prácticas hospitalarias, donde tendrán su primer contacto con pacientes quechua-hablantes y posteriormente durante su primer ejercicio profesional en el Serums ♣
